# Contemporary Biomarkers for Renal Transplantation: A Narrative Overview

**DOI:** 10.3390/jpm13081216

**Published:** 2023-07-31

**Authors:** Dorin Novacescu, Silviu Constantin Latcu, Razvan Bardan, Liviu Daminescu, Alin Adrian Cumpanas

**Affiliations:** 1 Doctoral School, Victor Babes University of Medicine and Pharmacy Timisoara, Eftimie Murgu Square, No. 2, 300041 Timisoara, Romania; novacescu.dorin@umft.ro; 2 Department of Urology, “Pius Brinzeu” Timisoara County Emergency Hospital, Liviu Rebreanu Boulevard, Nr. 156, 300723 Timisoara, Romania; razvan.bardan@umft.ro (R.B.); daminescu75@gmail.com (L.D.); cumpanas.alin@umft.ro (A.A.C.); 3 Department XV, Discipline of Urology, Victor Babes University of Medicine and Pharmacy Timisoara, Eftimie Murgu Square, No. 2, 300041 Timisoara, Romania

**Keywords:** organ/renal/kidney transplantation, precision/personalized medicine, diagnostic/predictive non-invasive biomarkers, immunopathology of acute allograft rejection (AR), chronic allograft dysfunction (CAD), glomerular vs. tubular nephron damage, clinical operational tolerance (COT), delayed graft function (DGF), ischemia–reperfusion injury (IRI)

## Abstract

Renal transplantation (RT) is the preferred treatment for end-stage renal disease. However, clinical challenges persist, i.e., early detection of graft dysfunction, timely identification of rejection episodes, personalization of immunosuppressive therapy, and prediction of long-term graft survival. Biomarkers have emerged as valuable tools to address these challenges and revolutionize RT patient care. Our review synthesizes the existing scientific literature to highlight promising biomarkers, their biological characteristics, and their potential roles in enhancing clinical decision-making and patient outcomes. Emerging non-invasive biomarkers seemingly provide valuable insights into the immunopathology of nephron injury and allograft rejection. Moreover, we analyzed biomarkers with intra-nephron specificities, i.e., glomerular vs. tubular (proximal vs. distal), which can localize an injury in different nephron areas. Additionally, this paper provides a comprehensive analysis of the potential clinical applications of biomarkers in the prediction, detection, differential diagnosis and assessment of post-RT non-surgical allograft complications. Lastly, we focus on the pursuit of immune tolerance biomarkers, which aims to reclassify transplant recipients based on immune risk thresholds, guide personalized immunosuppression strategies, and ultimately identify patients for whom immunosuppression may safely be reduced. Further research, validation, standardization, and prospective studies are necessary to fully harness the clinical utility of RT biomarkers and guide the development of targeted therapies.

## 1. Introduction

Renal transplantation (RT) is currently the optimal treatment option for patients with end-stage renal disease (ESRD), providing survival benefits, improved health-related quality of life, and cost-effectiveness, compared to dialysis [1]. Despite significant improvements in immunosuppressive therapies and surgical techniques, pervasive challenges still remain unaddressed regarding the complex, multi-modal, clinical management of RT patients. These challenges include early detection of graft dysfunction, timely identification of rejection episodes, personalization of immunosuppressive therapy, and prediction of long-term graft survival. Recently, biomarkers have emerged as valuable tools in addressing these challenges, offering the potential to revolutionize the clinical management of RT patients.

Recent advancements in immunosuppressive therapy have reduced acute rejections (ARs) and improved short-term renal allograft half-life [2]. Even so, late allograft loss still constitutes a major clinical issue post RT [3]. Current monitoring of renal allograft function relies upon serum creatinine measurement and needle-core renal biopsy, both of which have limitations. Creatinine levels rise only in later stages of allograft injury and cannot differentiate between specific injury types or predict chronic injury progression. Needle-core renal biopsy, though considered the gold standard, is invasive, cannot be safely performed repeatedly to monitor injury progression, has potential sampling biases, carries a 1–2% risk of significant complications, and its overall predictive power is poor in RT patients [4,5]. In fact, molecular-level tissue sample examination can detect immune response abnormalities before they become histologically evident [6]. The development of non-invasive, reliable, and predictive biomarkers for early diagnosis and monitoring of clinical conditions post-RT is essential for personalized treatment.

Biomarkers represent measurable objective indicators of normal biological processes, pathogenic visceral responses, and/or therapeutic interventions [7], and thus may also provide critical information about the state of the transplanted organ, i.e., the kidney allograft. Assays for proteomic, metabolomic, transcriptomic, and genomic biomarkers, derived from various biological sources, i.e., donor/recipient peripheral blood/serum/lymphocytes or urine, and tissue biopsy specimens have been extensively explored due to their notable clinical potential in RT, namely, to monitor allograft function, detect early rejection, guide immunosuppressive treatments, and predict long-term allograft survival and RT patient outcomes. The inclusion of validated gene transcripts/classifiers in the Banff classification for rejection highlights the growing importance of biomarkers in post-RT pathology [8]. Thus, it is becoming increasingly clear that further integration of these emerging biomarkers into clinical practice could significantly improve patient care and potentially optimize RT outcomes.

Generally speaking, biomarkers could, at least in theory, play a host of essential clinical roles throughout each step of the entire RT process [4], namely: (1) preoperative donor assessment and kidney allograft retrieval—prediction of short-term outcomes/risk of postoperative complications, i.e., delayed graft function (DGF); (2) in the perioperative setting—assessment, identification and characterization of subacute and/or AR processes, thus enabling more timely interventions; (3) postoperatively, for the crucial differential diagnosis between true chronic rejection (CR) vs. chronic allograft dysfunction (CAD)—similar clinically, yet require completely different treatments, with CR being immunologically mediated, whereas CAD is usually the result of various non-immunological pathogenic factors; (4) long-term monitoring of allograft injury occurrence [4]. Furthermore, biomarkers associated with RT patient immune tolerance are also highly coveted and of great importance for clinical management, as they could potentially allow for the progressive tapering or even complete discontinuation of postoperative immunosuppression, thus further reducing the risk of treatment-associated side effects and complications.

Beyond specific clinical context, RT biomarkers can be classified based on their individual capacity to assess immunological vs. non-immunological outcomes. Immunological outcomes are primarily related to rejection and immune tolerance, whereas non-immunological outcomes are mainly related to tissue injury [9]. Conversely, regarding nephron targeted biomarkers, a further classification based on individual histological nephron component specificity, i.e., glomerular vs. tubular, may also prove useful for the better characterization of pathogenesis and a more nuanced understanding of non-specific patient manifestations [10]. However, non-invasive biomarkers are indeed the primary candidates for clinical application in RT management, due to their inherent practicality, ease of assessment and minimal patient discomfort. Promisingly, non-invasive assessments for RT patients currently include: messenger (m)RNA transcripts; lymphocyte phenotype markers; chemokines; micro(mi)RNA; and donor-specific antibodies (DSA), i.e., antibodies that react specifically to antigens from the organ donor [4].

Notwithstanding the potential benefits of RT biomarkers, their clinical application is not without challenges. Overall, the actual utility of RT biomarkers in real-life patient management is largely dependent on their individual evaluation metrics, such as: sensitivity; specificity; positive predictive value; negative predictive value; receiver operating characteristics (ROC) curves. These metrics help determine the biomarker’s precision and reliability in identifying a condition or predicting an outcome, which are critical for guiding clinical decisions [11,12]. Moreover, validation of biomarker assay results can be affected by inter-observational variability (differences in results between evaluators) and inter-laboratory or inter-platform methodological heterogeneity (differences in results due to variations in laboratory methods or testing platforms). These can create discrepancies in biomarker measurements, limiting their predictive power and possibly leading to result misinterpretation [13]. Therefore, before new biomarkers can be confidently integrated into clinical practice, they must undergo thorough validation studies and assay standardization. Validation studies test the biomarker in a large, diverse patient group to ensure its accuracy and reliability across different clinical scenarios. Assay standardization ensures the methods used to detect/measure the biomarker are consistent and reproducible, providing dependable results regardless of where or when the test is conducted.

In summary, while biomarkers hold significant potential for improving patient outcomes in RT, their development, validation, and application involve careful consideration of various factors, including their predictive accuracy, ease of measurement, and consistency across different individuals and testing environments. As research in this area continues, the aim is to overcome these challenges and harness the full potential of biomarkers in guiding personalized care for RT patients, with scientists and physicians seeking non-invasive ways to detect allograft issues early and guide management and prognosis of both allograft and patient outcomes.

The objective of this review is to provide a comprehensive analysis of the contemporary clinical applications of biomarkers in the management of RT patients, focusing on post-RT non-surgical allograft complications. By synthesizing the currently available scientific medical literature, we aim to shed light on the most promising biomarkers, their main biological characteristics and their potential roles in improving clinical decision-making and RT patient outcomes.

## 2. Immunopathology of Nephron Injury and Allograft Rejection

The recent clinical introduction of more potent immunosuppressive drugs has resulted in a decreased incidence of AR. Nonetheless, about 10% of kidney transplant recipients still experience an AR episode within the first year after RT [14]. Although these episodes can generally be treated with intravenous steroids and/or anti-thymocyte globulin, their occurrence can have a negative impact on graft outcome. Routine immunologic laboratory tests are already being used to determine a patient’s immunologic sensitization and to assess the risk of adverse graft outcomes. The complement-dependent cytotoxicity test, performed pre-RT, has significantly reduced the incidence of hyper-acute rejection [15]. Similarly, pre-RT human leukocyte antigen (HLA) alloantibody screening aids in optimizing donor selection. Post-RT HLA alloantibody screening assists in identifying the specific type of AR and potential antibody impact on graft function [16].

In fact, AR episodes, which are most prevalent in the first few weeks after transplantation, can be categorized into T-cell-mediated rejection (TCMR) and antibody-mediated rejection (ABMR) [17]. Essentially, TCMR involves lymphocyte infiltration and proliferation within the interstitial space of the kidney allograft, which will the subsequently induce cytotoxic effects on renal tubular epithelial cells, causing inflammatory responses, i.e., “tubulitis”. Similarly, vascular rejection, a more severe variant of TCMR, involves mononuclear cells invading arteries, leading to arteritis and potentially severe transmural necrosis of allograft vasculature. Conversely, in ABMR, DSA target HLAs or non-HLAs on the donor endothelium, leading to antibody-dependent cellular cytotoxicity and complement activation [16].

DSA refer to the antibodies that a transplant recipient forms against specific HLA antigens found on the donated organ. These antibodies can inflict allograft nephron damage, by inducing multi-lamination of the peritubular capillary basement membranes, or arteriopathy that manifests as intimal fibrosis [18]. The DSA endothelial cell injury can trigger platelet aggregation and leukocyte recruitment, potentially leading to graft failure. Thus, when the allograft is subjected to rapid surges in high-titer DSA, AR occurs, usually either in sensitized recipients, or as de novo responses in non-sensitized patients who do not strictly adhere to their immunosuppressive treatment. Alternatively, CR mediated by DSA occurs in response to a slower emergence of these antibodies, which can be of high or low titer and may be either transient or persistent [18].

While substantial research is being conducted to develop therapeutic strategies aimed at reducing DSA levels [19], our current understanding of how to prevent the initial formation of DSA is still limited. Moreover, risk factors associated with DSA development are not fully defined. Early evidence suggests that specific immunosuppressive treatments could influence DSA formation [20]. Specifically, it appears that treatments based on calcineurin inhibitors are less likely to be associated with DSA formation compared to those based on mTOR inhibitors or lower mycophenolic acid levels [21].

Clinically, microcirculation lesions, C4d deposition in peritubular capillaries, and the presence of DSA in the patient’s serum suggest ABMR. However, DSA can be identified in the serum of RT recipients many years before any signs of clinical graft dysfunction appear. Hence, it is crucial to routinely monitor DSA in the follow-up of transplant recipients, even though uniform protocols are not yet in place [22]. Moreover, the onset of de novo DSA (dnDSA) post-RT has been firmly linked to poor graft outcomes in adults as well as children [22,23]. The formation of dnDSA, in general, is associated with lower 10-year graft survival rates, even in pediatric studies [22]. Managing the aftermath of chronic (c)ABMR is typically even more challenging. Given this information, dnDSA are recognized as reliable biomarkers that can predict late acute ABMR, cABMR, transplant glomerulopathy, and graft loss [24]. Even so, their clinical significance is contingent upon certain characteristics of the antibody itself, such as its IgG subclass, which affects its capacity to bind complement cascade components and engage effector cells through Fc receptor binding. For instance, IgG3 subclass dnDSA can bind to complement component (C)1q more efficiently, activate the classical pathway of the complement cascade, and often lead to acute ABMR, whereas IgG4 DSA, which cannot bind Cs, primarily operate through the Fc receptor to magnify alloresponses [25].

**Figure 1:**
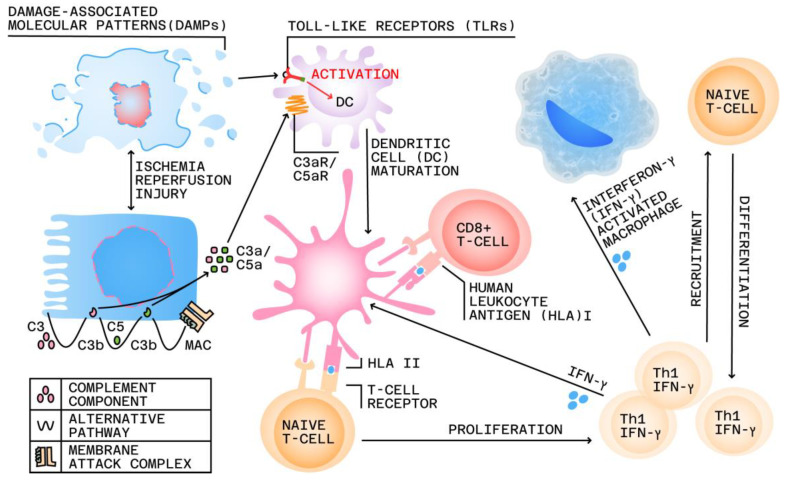
The pathogenic role of innate and adaptive immunity in renal allograft damage. Adapted from Eikmans et al. [16].

Indeed, as detailed in Figure 1, the transplant recipient’s adaptive immune system plays a central role in allograft TCMR. Thus, within this process, alloreactive T-lymphocytes, which represent between 1and 10% of T-lymphocytes overall, interact with mismatched HLAs on donor-derived antigen-presenting cells (APCs) [26]. This interaction, known as direct allorecognition, and the subsequent interaction between recipient APCs and CD4+ T cells, known as indirect allorecognition, promote T cell proliferation and differentiation [27]. Activated CD8+ T cells release perforin and granzyme B, which induce apoptosis of target cells [28], while monocytes and myeloid dendritic cells (DCs) infiltrate the graft and contribute to AR [29,30]. However, innate immunity also plays a role in transplant injury, via intra-allograft complement cascade activation.

Normally, the innate immune system provides a general defense against foreign pathogens by employing the complement system and cellular responses from macrophages and DCs. These cells possess Toll-like receptors (TLRs) that can identify pathogen-related molecular patterns on invading microbes [16]. Importantly, post RT, ischemia reperfusion injury (IRI) is, at least to a certain degree, virtually unavoidable, due to the inherent conceptual and methodological limitations of contemporary surgical strategies [31]. Thus, as detailed in Figure 1, the process of post-RT allograft TCMR damage is initiated by this pervasive associated mechanism of IRI, which determines tubular cellularity apoptosis, causing the subsequent release of damage-associated molecular patterns (DAMPs). These post tubular injury DAMPs, typically concealed within healthy cells, will then bind to TLRs on DCs, triggering their activation and maturation [32,33]. Furthermore, IRI can also lead to local activation of the complement cascade. The DCs present donor-derived human leukocyte antigen (HLA) target epitopes and co-stimulatory molecules to naïve T-cells, leading to the differentiation of these cells into interferon (IFN)γ-producing T-helper (Th)1 cells. This, in turn, will further stimulate the maturation of other additional recipient DCs, induce macrophage activation and recruitment, and direct the differentiation of CD8+ T-cells. Concurrently, IRI can also induce a local increase in complement component 3 (C3). When C3 is cleaved by the alternative pathway, C3b is deposited on cellular membranes, instigating the activation of the complement cascade. The breakdown of C3 leads to the release of small fragments, i.e., C3a and C5a, during complement activation, both of which have pro-inflammatory effects. The subsequent formation of the membrane attack complex (MAC) results in lysis of the targeted cell and further release of DAMPs [16].

## 3. Glomerular vs. Tubular Biomarkers for Allograft Nephron Damage Assessment

Following RT surgery, the kidney allograft may either immediately resume normal functionality or experience a delay of several days or even weeks, i.e., DGF. A lack of normal kidney transplant function can lead to acute kidney injury (AKI) [34,35], nephrotic syndrome (NS) [36,37], and aggravation of pre-existing chronic kidney disease (CKD) [38]. Thus, post RT, it is crucial to monitor specific biomarkers that can detect disease progression and identify which kidney functions are at risk, facilitating the prompt implementation of appropriate treatments [39,40,41]. The administration of immunosuppressants to prevent renal graft rejection can, ironically, lead to progressive renal tissue damage (such as interstitial fibrosis, tubular micro calcifications, and renal tubule atrophy), due to the high toxicity of these drugs. The majority of renal pathological changes affect the glomeruli, proximal and distal tubules, as well as the vascular endothelium.

**Figure 2:**
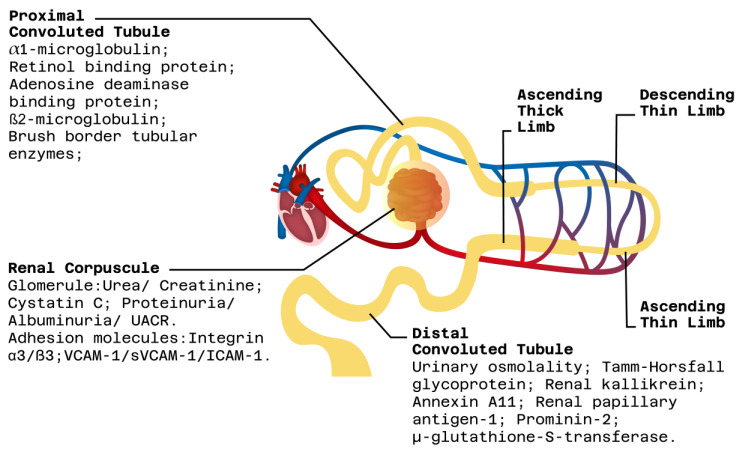
Classification of conventional post-RT allograft injury biomarkers, according to nephron component specificity [10].

Renal proximal tubular cells (Figure 1), which have the highest metabolic activity and contain large amounts of mitochondria, lysosomes, and peroxisomes, are typically the first to suffer damage. Other sections of the nephron, such as Henle’s loop, distal tubules, and collecting tubules, usually sustain damage later on. There are many biomarkers available to identify injury in different areas of the renal nephron, such as the glomerulus, or the proximal and distal tubules [10]. In Figure 2, we provide a schematic summary of current conventional non-invasive clinical biomarkers, which have intra-nephron specificities, i.e., glomerular vs. tubular (proximal vs. distal).

All in all, scientific advancements in molecular biology, i.e., novel genomic, transcriptomic, proteomic, and metabolomics experimental data, have revealed an array of new, nephron-segment-specific, post-RT biomarkers for allograft damage. There are high hopes for proteins that present nephron specificities or are locally produced at the site of nephron damage. Traditional biomarkers, particularly enzymuria, still hold diagnostic value in assessing renal tubule function. While this abundance of biomarkers, in particular, may in fact reflect that their individual diagnostic value may be limited, the search for a universal integrative biomarker for allograft assessment remains challenging. Instead, identifying putative biomarker proteins useful in diagnosing key allograft disease features is likely to yield better results [10].

In Table 1, we provide additional data regarding the classification, definitions and currently available supporting data for these nephron-component-specific biomarkers, in hopes of providing clinicians with additional useful evidence regarding the early detection of nephron damage post RT.

Multiple promising biomarkers for kidney damage have been identified, with the most relevant and best-studied being neutrophil gelatinase-associated lipocalin (NGAL), CYC, kidney injury molecule-1 (KIM-1), β2M, and interleukin-18 (IL-18) [86]. Notably, in kidney allograft recipients, urinary KIM-1 expression provides prognostic information related to the rate of renal function decline, regardless of the underlying kidney pathology [87]. However, validation of these kidney markers in various pathological conditions is still ongoing. High diagnostic value is still held by certain enzymes in diagnosing renal diseases, such as HEX and its isoenzyme HEXB as markers of proximal tubular damage, AAP or GST as markers of the tubular brush border membrane, and cytosolic FBP-1,6 for assessing graft function [10]. A panel of urinary proteins and enzymes may serve as a practical marker for evaluating the nephron function of a transplant kidney and prognosticating the renal allograft’s fate. Future biomarker discoveries and research techniques may change the practical approach to treating patients with renal grafts.

## 4. Biomarkers for Non-Surgical Renal Allograft Complications

Postoperative monitoring of RT patients is a critical aspect of care management [88]. Currently, the standard of care recommended is quarterly measurements of urinary protein excretion, within the first year. Moreover, screening for viral infections, i.e., Polyoma and/or Epstein–Barr virus, using plasma nucleic acid testing, should be done monthly, for at least the first three months post RT, and then every three months, until the end of the first year. A percutaneous renal allograft needle biopsy is necessary if there is an unexplained rise in serum creatinine. The Banff classification system provides standardized criteria for histological diagnosis of AR, scoring inflammation in various renal compartments [8]. However, changes in serum creatinine are not specific to graft injury: variations might indicate an intrinsic renal process like AR or graft infection, or a transient process such as the hemodynamic effects of calcineurin inhibitors or pre-renal volume depletion [88]. AR involves various stages, with clinical signs of graft damage appearing late, following a period of subclinical graft damage [89,90]. Thus, serum creatinine levels may remain unchanged despite significant kidney injury.

Moreover, biopsies can also lead to complications for the transplant recipient [91], and being an in-patient procedure, can be quite costly. Other drawbacks of allograft biopsy include potential sampling errors and/or differences in interpretation among pathologists [92]. Therefore, there is a pressing need for alternative, less invasive, yet more sensitive, post-RT biomarkers for diagnosing acute graft rejection, i.e., subclinical allograft nephron damage. Discovering and validating biomarkers that correlate with and/or can predict AR early on, thus capable of enhancing the objectivity, accuracy and overall efficacy of therapeutic decision making for clinicians, are high priorities among most ongoing RT research initiatives [13]. Through regular sampling, the development of rejection might be predicted before tissue injury actually develops. Biomarker information could also help differentiate high-risk patients from low-risk ones, facilitating individualization of immunosuppressive drug therapy.

### 4.1. Acute Allograft Complications

Post RT, the transplanted renal allograft may be vulnerable to several acute insults, including immunologic injury, IRI, medication related nephrotoxicity, and surgical complications [93]. In the acute context, IRI, in particular, represents, to some degree, an inevitable postoperative occurrence following RT, and can have an impact on both short-term and long-term allograft outcomes [4].

#### 4.1.1. Delayed Allograft Function

The clinical consequences of IRI may include DGF and allograft rejection, i.e., AR, CR, and/or CAD [94]. The severity of IRI is influenced by various donor/recipient-specific factors, as well as associated organ storage conditions [95]. The utilization of extended eligibility criteria for donors and of organs from deceased donors, increases the risk of severe IRI [4]. It is crucial to understand the factors that contribute to severe IRI in order to assess the risk to recipients and diagnose IRI promptly. This enables the implementation of preventive and treatment measures, to diminish the subsequent DGF and prevent rejection. The identification of biomarkers for IRI and IRI derived DGF can aid in these efforts.

Several molecules, indicating allograft tubular and/or vascular damage, have demonstrated associations with the occurrence and severity of IRI [4]. In turn, the severity of IRI influences the occurrence of DGF [96], with graft survival being closely linked to the occurrence of DGF [97]. In Table 2, we provide a summary of the currently available evidence regarding the use of contemporary IRI/DGF-associated biomarkers in various clinical settings.

#### 4.1.2. Acute Allograft Rejection

To this day, AR still constitutes a major cause of early allograft loss and remains a significant clinical hurdle in post-RT patient management. Current gold-standard methods for diagnosing AR rely on histological examination of renal allograft biopsies, which are invasive and subject to sampling variability. Therefore, numerous studies have focused on identifying non-invasive biomarkers that can predict, preoperatively, the risk of AR occurrence later on, and/or accurately detect AR postoperatively, thus potentially reducing the need for allograft biopsies.

In the pre-transplant setting, serum biomarkers have mainly been explored for their potential to predict AR. Most investigated among them, soluble CD30 (sCD30), is a glycoprotein found on human CD4+/CD8+ Th lymphocytes, that produce Th2-type cytokines [118]. sCD30 helps identify recipients who may generate an immune response against a transplanted kidney, acting as a predictor of poor graft outcomes [119], often due to a higher incidence of AR [120,121,122,123,124]. Conversely, Th1 immune response is associated with IFN-γ-producing cells and IFN-induced chemokines, i.e., C-X-C motif chemokine ligand (CXCL) 9/10. Several studies have found that the pre-RT frequency of donor-specific IFN-γ-producing cells correlates with AR among recipients of cadaveric kidney allografts [125,126,127,128]. Increased serum levels of CXCL10 in recipients have been linked to higher transplant failure due to increased AR incidence [129,130].

As reported in Table 3, post-RT urinary CXCL9 mRNA levels were found to be predictive of AR, with lower levels indicative of low risk for immunological events [131,132]. Several urinary biomarkers were correlated with post-RT allograft injury, including CXCL9, CXCL10, C-C motif chemokine ligand 2 (CCL2), NGAL, IL-18, CYC, KIM1, and T-cell immunoglobulin/mucine domains-containing protein 3 (TIM3) [133]. Urinary CXCR3 chemokine receptor is emerging as a promising candidate for detecting subclinical inflammation [134]. Furthermore, certain genes in peripheral blood lymphocytes and kidney graft biopsies have been shown to identify patients with AR. These genes relate to immune inflammation, transcription factors, cell growth, and DNA metabolism. Moreover, T lymphocytes and IFNγ-producing Th1 cells are being studied as cellular markers of AR [135,136]. Finally, donor-derived cell-free (ddcf)DNA has been detected in the recipient’s blood and urine during AR episodes [137,138].

Overall, as is the case for emerging biomarkers in contemporary renal oncology [164,165,166,167,168,169], while numerous potential AR biomarkers have been identified through the plethora of recent studies published, their specificity and sensitivity in clinical practice remains to be determined. For instance, ddcfDNA has been found to be increased in confirmed AR cases [138], yet it is also present in other kidney injuries, such as pyelonephritis, making it less specific as a marker for AR [170]. Similarly, the kidney solid organ response test (kSORT) has shown high sensitivity in predicting AR and subclinical AR, but these findings need further validation [157,160]. The common rejection module (CRM), a set of 11 genes found to be overexpressed in AR across different organ transplants, is another promising development, but again, further studies are needed to validate the existing results and determine their clinical utility [161,162,163]. Most recently, capitalizing on longstanding, sustained scientific efforts aimed at the molecular characterization of the mechanisms involved in graft rejection after solid organ transplantation, the Molecular Microscope Diagnostic System (MMDx), i.e., a clinical tool which uses mRNA to differentiate between specific AR subtypes, has been investigated in the context of RT. A 77% agreement with histology was shown for TCMR and ABMR, and a 76% agreement for no rejection, when assessed without prior knowledge of histology and HLA profiles. Interestingly, the MMDx showed an 87% agreement with clinical judgment, which is higher than the agreement with histology at 80%. This suggests that the MMDx may offer additional or more nuanced information beyond traditional histology in diagnosing transplant rejection [171].

### 4.2. Chronic Allograft Rejection vs. Dysfunction

Even though, broadly speaking, the field of RT patient management has seen significant sustained advancements throughout recent decades, the progress achieved was inhomogeneous, i.e., persistently disproportionate outcomes and incidence rates between early acute complications and latent chronic allograft dysfunction. Overall, long-term renal allograft survival rates have been notably lagging behind, as opposed to the ever increasing short-term (1 year) renal allograft survival rates currently reported, and the significantly declining occurrence of AR [3]. As postoperative survival intervals increase, the primary reason for latent renal allograft loss in post-RT patients is a clinical condition known traditionally as Chronic Allograft Nephropathy (CAN), characterized by the gradual non-specific deterioration of kidney transplant function. Despite numerous efforts, CAN’s origins remain complex and unattributable to a single cause. This appears to result from a variety of interconnected processes between the host and the transplanted organ, leading to ongoing kidney tissue damage, through both immune and non-immune-mediated mechanisms [172].

Thus, in recent times, CAN has been replaced clinically with the more modern, wider term chronic allograft dysfunction (CAD), facilitating the more accurate identification of true CR cases and allowing a finer distinction between immunological CR and other non-immunological causes of chronic dysfunction, such as drugs and viruses. The most recent genomic and proteomic data highlight the similarity in molecular injury patterns between AR and CAN. There is a so-called “threshold effect” for AR, and during its clinical phase, the molecular injury mirrors what is observed in CAN, albeit at a more intense level. Conversely, the continuous, low-grade immune activation in allograft tissues increases gradually post RT, independently driving the progression of CAN, without requiring overt AR episodes [4]. These findings are further validated by urinary proteomic studies [14,173].

Similarly, from a morphopathology perspective, CAN’s clinical manifestation has been redefined and renamed as Interstitial Fibrosis and Tubular Atrophy (IFTA) of unknown origin [174,175]. Histological examination of biopsies shows that IFTA occurs in ~50% of renal allografts, at 1-year post RT, ~70% at 2 years, and virtually all cases after 10 years [176,177]. In corroboration, further data clearly demonstrate a correlation between IFTA’s progression and renal function decline [178,179]. However, IFTA’s progression is not always linear or predictable, suggesting that aspects of the condition are dynamic. Consequently, there is an urgent need for developing new strategies to disentangle the intricate mechanisms of tissue injury that culminate in the development of CAN/IFTA, allowing for the identification and clinical implementation of a sensitive and reliable biomarker, or panel of biomarkers, able to distinguish AR from other forms of CAD.

In fact, current proteomic data suggest that non-invasive biomarkers may soon play a crucial clinical role in the identification of chronic allograft injury (CAI) and CR post RT. In order to identify relevant biomarkers for CAD, a plethora of proteomic investigations, using variable research platforms, on tissue biopsy, peripheral blood and, most frequently, urinary samples, have already been conducted, analyzing thousands of potential targets [172,180]. Even so, for the most part, these large-scale proteomic efforts have thus far failed to offer reliable genomic validation for the wide array of potentially impactful CAD-specific biomarkers identified, mainly due to inherent study design limitations. For instance, the study conducted by Quintana et al. [180] relied on single urine samples from 50 subjects: 32 CAD patients (14 with IF and 18 with chronic active-antibody-mediated rejection—caABMR) and 18 controls (8 stable post-RT patients and 10 healthy individuals). Even though >2000 protein signals were assessed, using modern mass spectrometry (MS), and subjected to unsupervised hierarchical cluster analysis, only 14 protein signals were reported as capable of distinguishing between samples from patients with IF vs. true CR, i.e., caABMR. However, these 14 protein ions were only identified by their mass/charge ratio and no further attempt was made to identify the actual proteins. Shortly after, the same author, using a different MS platform, reported on 6000 polypeptide ions assessed in post-RT urinary samples, i.e., 39 CAD patients vs. 32 controls, and found specific uromodulin and kininogen-1 derived peptides were notably more abundant in controls than in CAD patients, marking them as potential diagnostic biomarkers for CAD [181].

Thereafter, in an effort to find urinary proteomic profiles that could predict and distinguish/stratify CAD/IFTA, similar proteomic approaches, derived from the same conceptual premises, i.e., MS assessment of post-RT urinary samples, have been further explored fruitfully. Among urinary samples from 70 post-RT cases, 34 with confirmed IFTA vs. 36 controls with normal renal function, a 11.7 kDa protein, identified as β2M, has emerged as a highly reliable IFTA detection/screening/diagnostic biomarker, i.e., consistently ↑ β2M urinary levels in the confirmed IFTA cohort vs. control [182]. Corroborating these findings, in a seminal study, with a limited cohort (36 cases in total), 2D Fluorescence Difference Gel Electrophoresis (2DE-DIGE) managed to establish the normal urinary proteomic map of stable post-RT patients, while also identifying 21 potential urinary biomarkers, specific for different stages of IFTA, such as: A1AT, α1-B-glycoprotein, angiotensinogen (AGT), anti-TNFα antibody light chain, β2M, brevin, heparin-sulfate proteo-glycan, leucine-rich α2-glycoprotein-1 (LRG1), and transferrin [183].

Moreover, in a very recent investigation of urinary proteomics focused specifically on caABMR-specific biomarkers, urinary extracellular vesicle (EV) changes were assessed, using a combined approach, i.e., label-free liquid chromatography and tandem MS, with Western blot confirmation, in post-RT patients (26 cases with confirmed caABMR, 57 with long-term allograft survival and 10 rejection-free controls). After selecting only high-significance proteins, i.e., with a fold-change ≥ 1.5, the study reported six proteins, i.e., apolipoprotein A-1 (APOA1), zinc-α2-glycoprotein (AZGP1), ceruloplasmin (CP), hemopexin (HPX), polymeric immunoglobulin receptor (PIGR), and transthyretin (TTR), as potential biomarkers for caABMR, able to discriminate between caABMR vs. long-term allograft survival subgroups. Among these proteins, AZGP1 showed specificity for caABMR and was distinguishable from the rejection-free control group, with matching age at transplant, time since transplantation, and graft function [184].

In fact, currently, by analyzing pooled urinary proteins from AR, BKVN, and CAN cohorts, in comparison to stable transplant urinary samples, while using control fold-change criteria of >1.5 for each transplant injury phenotype (AR vs. BKVN vs. CAN/IFTA), proteomic analysis of post-RP patients has already managed to reveal specific proteins associated with each condition, potentially aiding differential diagnosis immensely [144]. As previously noted (see Table 3), in patients with AR, increases in ACTβ, DPP4, FGA, FGB, FGG, HIST1H4B, HLA-DRB1, KRT7, and KRT14 proteins were found. For BKVN, there were increases in Complement Factor H Related 2 (CFHR2), Family with Sequence Similarity 3 Member C (FAM3C), Histone Cluster 1 H2B Family Member A (HIST1H2BA), KRT8, KRT18, KRT19, KRT75, Ribosomal Protein L18 (rPL18), Stathmin1 (STMN1), Small Ubiquitin-like Modifier 2 (SUMO2). Lastly, in the case of CAN patients, increased levels of AGT, Calreticulin (CALR), Dystroglycan 1 (DAG1), FABP4, Family with Sequence Similarity 151 Member A (FAM151A), FAM3C, KIT Ligand (KITLG), LRG1, Lumican (LUM), and Serpin Family A Member 2 (SERPINA2P) were observed [4,144]. These specific proteins can therefore be considered as potential discriminatory proteomic biomarkers for different types of transplant injury phenotypes. However, further investigation and clinical trials would be required to confirm these findings and to evaluate their practical utility in a clinical setting.

Conversely, a particularly promising target discovery investigational strategy appears to be the combination of proteomics and genomics, i.e., proteogenomics [172,185]. Herein, a proteogenomic approach by Kurian et al. focused on discovering CAN/IFTA-specific biomarkers, in peripheral blood samples, collected from 77 post-RT patients, with significant clinical differences among themselves, in the hopes of developing a practical model for post-RT monitoring, based on serial, prospective measurements of the identified target signatures, throughout the lifespan of the renal allograft. Ultimately, this development would allow for optimal immunosuppressive drug management, with the potential to introduce personalized medicine to RT. Several hundred mRNAs and proteomic biomarkers were identified as potentially useful in the differential diagnosis and clinical staging of IFTA. Specifically, 302 proteins unique to mild CAI and, respectively, 509 proteins unique to moderate/severe CAI were reported. Despite the diversity and heterogeneity of patient samples, the predictive accuracy of these biomarkers was quite high, i.e., 80% for mild CAI vs. 92% for moderate/severe CAI [185].

Recent studies have utilized molecular tools such as miRNAs and gene expression analyses to better understand CAD/IFTA. One study identified five specific miRNAs (miR142-3p, miR-32, miR204, miR-107, and miR-211) that were differentially expressed in both allograft tissue biopsies and urine samples of post-RT patients affected by IFTA vs. control [186]. Another set of miRNAs (miR99a, miR-140-3p, mi 200b, and miR-200) were found to be differentially expressed at different time points post RT in relation to graft outcome, being useful for monitoring [187]. Notably, urinary miRNA profiles varied in IFTA patients based on whether they received a kidney from a living or cadaveric donor [188]. Furthermore, relevant for monitoring kidney allograft function in patients affected by IFTA, a study on IFTA renal biopsies showed significant upregulation of miR-142-5p and miR-142-3p, and downregulation of miR-211, as compared to controls (stable graft) [189]. Interestingly, the same results were observed in peripheral blood cells from the same IFTA cohort, suggesting that peripheral blood cells could provide an additional non-invasive method for monitoring graft function [189]. Lastly, in another post-RT study, miR-486-5p was found to be significantly over-expressed in patients who produced DSA and/or had biopsy-confirmed caABMR [190]. This suggests that specific miRNAs might serve as potential biomarkers for graft rejection. The discovery of these miRNA profiles in different patient groups suggests a promising avenue for non-invasive diagnosis and monitoring of post-RT complications.

Genomic studies have also uncovered promising evidence regarding specific CAI biomarkers. Microarray analysis has identified upregulation of 10 genes (fold-change >6.00) related to fibrosis, extracellular matrix deposition, and immune response, in renal allograft tissues of 11 patients with biopsy confirmed CAD/IFTA, as compared to controls [191]. Using PCR, the markers identified through the microarray analysis in these CAD/IFTA patients, such as transforming growth factor beta (TGF-β), epidermal growth factor receptor (EGFR), and AGT, were examined in urinary/peripheral blood samples, retrieved at the time of biopsy, and shown to be statistically different in urinary, but not in blood samples, when compared to controls [191]. On a larger scale, in the aforementioned CTOT-04 trial, besides the validated three-gene signature (CD3ε mRNA, CXCL10 mRNA, and 18S rRNA) predictive of AR (see Table 3) [149], an additional four-gene signature (vimentin, NKCC2, E-cadherin, and 18S rRNA) in urinary mRNA was reported as diagnostic for IFTA, providing a potential non-invasive biomarker for this condition [192]. Similarly, within another computational gene expression score, the tCRM, a subset of seven genes (CD6, INPP5D, ISG20, NKG7, PSMB9, RUNX3, and TAP1) demonstrated a higher predictive value for the development of IFTA over time [162].

Conversely, the international Genomics of Chronic Allograft Rejection (GoCAR) study, a prospective microarray analysis of gene expression profiles in allograft tissue samples from 159 RT recipients, with stable graft function at 3 months post RT, identified a set of 13 genes that were independently predictive of allograft fibrosis at 12 months after RT. This gene signature, i.e., Ankirin repeat and SOCS box-containing 15 (ASB15), Coiled-coil-helix-coiled-coil helix domain containing 10 (CHCHD10), Four jointed box 1 (FJX1), Kelch-like family member 13 (KLHL13), Kidney-associated antigen 1 (KAAG1), Met proto-oncogene (MET), Retinoid X receptor alpha (RXRA), Ring finger protein 149 (RNF149), Serine incorporator 5 (SERINC5), Sprouty homolog 4 (SPRY4), Suppressor of tumorigenicity 5 (ST5), TGF-β-induced factor homeobox 1 (TGIF1), and Wingless-type MMTV integration site family member 9A (WNT9A), was found to have a superior predictive value for allograft fibrosis development, outperforming clinical and pathological variables [193].

Lastly, a very recent meta-analysis of molecular datasets identified a robust distinctive transcriptional response in IFTA allografts, as compared to non-IFTA cases, i.e., 85-gene signature significantly associated with IFTA. In a novel approach, genomics was thereafter used to identify novel potential therapeutic agents for IFTA. Through computational repurposing analysis of the aforementioned 85-gene signature, besides validation of azathioprine, an already established treatment for AR and pulmonary fibrosis, two promising novel drugs were identified: Kaempferol, which attenuates TGF-β1, and Esculetin, which inhibits the Wnt/β-catenin pathway. Preclinical models demonstrated the effectiveness and safety of these drugs, suggesting their potential for therapeutic intervention in IFTA [194]. All in all, these studies highlight the significant potential of molecular tools for diagnosis, prognosis, and treatment of CAD/IFTA.

## 5. Immune Tolerance and Therapeutic Drug Monitoring

Drug level monitoring is an important biomarker for assessing the proper use of immunosuppressive drugs in transplant recipients. It is commonly performed for drugs such as tacrolimus, cyclosporine, everolimus, and sirolimus [195]. However, monitoring mycophenolic acid (MPA) using single-sample drug concentrations in the recipient’s blood immediately before the next dose is administered may not accurately reflect the overall drug exposure. To overcome this limitation, MPA area under the curve estimation has been introduced as a more effective clinical tool. However, it requires multiple concentration samplings, which can be less practical, especially in pediatric patients [195,196].

In the case of tacrolimus, intra-patient variability (IPV) refers to fluctuations in blood levels over time in individual patients receiving a fixed dose. High IPV of tacrolimus has been associated with the development of DSA, allograft dysfunction, rejection, transplant glomerulopathy, and late graft loss in adult studies [197]. In pediatric studies, tacrolimus IPV has been correlated with de novo DSA development, but its correlation with rejection, decline in graft function, and graft loss is weaker. This may be due to differences in defining cut-off values, cohort size, and methodological variations [198,199].

Future perspectives in drug monitoring advocate the use of expert systems to estimate drug exposure [200], the development of novel techniques for simultaneous evaluation of multiple drugs, and a shift towards the concept of “time in therapeutic range” [201]. This concept, already employed in other medical fields, can provide more precise predictors of under-suppression and the potential risk of allograft rejection. Advancements in drug monitoring techniques and the use of more comprehensive predictors of drug exposure hold promise for improving individualized immunosuppressive therapy and optimizing transplant outcomes.

Global immunosuppression markers are important for assessing the overall intensity of immunosuppression in transplant recipients. Albeit still subject to scientific scrutiny and clinical exploration, various techniques, including flow cytometry and pathogen-specific T-cell response assays, show promise, but still require further validation and standardization [14]. These biomarkers have the potential to improve individualized immunosuppressive therapy and identify patients who can safely reduce their immunosuppression levels. Simple numeric quantitative measurements of lymphocytes have not proven to be reliable indicators, even for determining the dosage of immunosuppressive agents used for depletion induction. AR can occur even in patients with profound T-cell depletion and without additional immunosuppression [202]. One potential measure of global immunosuppression is the quantification of CD4+ T-cell adenosine triphosphate (ATP) production after polyclonal antibody stimulation in vitro [203]. This assay has only been assessed in a non-controlled trial thus far, and still lacks validation and substantial evidence of its utility, yet it has been marketed commercially as a clinical tool for post-RT monitoring [14].

Indirect assessment of global immunosuppression can be performed by quantifying biomarkers of pre-existing protective immunity. Techniques such as PCR, enzyme-linked immunosorbent spot (ELISPOT), and flow cytometry have been developed to detect pathogen-specific T-cell responses against common viral pathogens like cytomegalovirus (CMV), Epstein–Barr virus, and BK virus [204,205,206]. However, these techniques are labor intensive and lack standardization across transplant centers. The detection of IFNγ production in response to CMV peptides, using currently available, well-validated CMV immune assays, i.e., ELISPOT and/or QuantiFERON, might help standardize monitoring for this viral infection [207,208], but further characterization of the correlations between immunosuppression degree, viremia risk, and allograft rejection risk is needed. Most recently, multiple potentially impactful, novel experimental applications for immune monitoring post RT have been developed, centered around the most abundant virus of the commensal human virome, the non-pathogenic Torque Teno Virus (TTV), i.e., an anellovirus that does not cause disease directly, but rather replicates based on the immune status of its host [209]. Thus, as TTV viremia has already previously been shown to correlate with the overall level of immunosuppression, while also predicting the occurrence of viral infections, graft rejection, and antibody response after COVID-19 vaccination in lung transplant recipients, it has now been proposed and investigated as a biomarker of functional immunity in RT patients [210]. Apparently, monitoring TTV viremia could be an additional tool for predicting CMV reactivation. However, while these TTV methods have potential in risk prediction, they have not been explicitly tested in drug titration protocols and have not clearly documented a direct drug-infection relationship [209,210].

Flow-cytometry-based assessment of lymphocyte phenotypes has been investigated as a means of gauging immunosuppression intensity. Interestingly, while T-cell phenotypes have not provided significant insights, three studies have observed a B-cell phenotype signature associated with spontaneously immuno-tolerant RT patients [211,212,213]. This unexpected association suggests that transplant recipients may have altered peripheral blood lymphocyte repertoires that warrant further investigation. If validated, an assay based on flow cytometry could be easily adopted in clinical laboratories to prospectively identify tolerant patients, allowing clinicians to reduce immunosuppression and avoid unnecessary adverse drug effects [14].

Even so, clinically stable allograft function, within acceptable parameters, under the long-term absence of immunosuppressive therapy, i.e., operational tolerance (OT), post RT, represents an exceedingly rare phenomenon, with only ~100 cases hitherto reported [214]. However, some studies have identified specific genes that are upregulated in OT patients. In different patient cohorts and using various microarrays, 39 genes were found to be elevated in OT, with 24 of them being B-cell related. CD79b and prepronociceptin were among the most highly expressed OT-related genes [211,212,215]. Furthermore, miR-142-3p was also found to be upregulated in B cells of OT patients [216].

Genomic studies have revealed gene expression changes associated with tolerance. Membrane-spanning 4-domains A1 (MS4A1/CD20), T-cell leukemia/lymphoma 1A (TCL1A), CD79b, tolerance-associated gene 1 (TOAG1), and FOXP3 genes were found to be upregulated in peripheral B cells [217]. A multicenter study reviewed a cohort of kidney transplant recipients to identify an immunosuppression-independent gene signature for predicting tolerance. They identified nine genes, including Ataxin 3 (ATXN3), BCL2-related protein A1 (BCLA1), Eukaryotic translation elongation factor 1 alpha 1 (EEF1A1), Gem-associated protein 9 (GEMIN7), Immunoglobulin lambda constant 1 (IGLC1), Membrane-spanning 4-domains A4A (MS4A4A), Nuclear factor of kappa light polypeptide gene enhancer in B cells inhibitor, alpha (NFκBIA), RAB40C-member of RAS oncogene family, and TNF, α-induced protein 3 (TNFAIP3) [218]. Additionally, the kidney spontaneous operational tolerance test (kSPOT) program identified 21 genes involved in OT [219]. Among them, Kruppel-Like Factor 6 (KLF6), Basonuclin 2 (BNC2), and Cytochrome P450 Family 1 Subfamily B Member 1 (CYP1B1) were used to develop a three-gene assay with high accuracy for detecting OT [213,219,220].

Overall, the pursuit of a tolerance signature in RT remains challenging due to the small number of OT patients. Biomarker studies are primarily focused on identifying OT in post-RT patients, i.e., screening applications. Various large-scale approaches, such as kSORT, tCRM, uCRM, and kSPOT, may assist in reclassifying transplant recipients based on immune risk threshold and determining which patients can benefit from immunosuppression withdrawal or minimization [14].

## 6. Conclusions

Biomarkers have emerged as valuable tools in addressing the challenges associated with the clinical management of RT patients. Despite improvements in immunosuppressive therapies, there are still pervasive challenges in early detection of graft dysfunction, timely identification of rejection episodes, personalization of immunosuppressive therapy, and prediction of long-term graft survival. Serum creatinine measurements and needle-core renal allograft biopsy, the current methods for evaluating allograft function, have important limitations. Serum creatinine levels are non-specific and unable to differentiate between specific types of injury, while renal allograft biopsy is invasive and cannot be performed repeatedly. Non-invasive biomarkers offer the potential to revolutionize the clinical management of RT patients by providing early diagnosis and monitoring applications of allograft function, i.e., timely detection of complications. Biomarkers associated with AR, CAD, and immune tolerance have shown promise in various studies. Serum and urinary biomarkers, as well as gene signatures and miRNAs, have been identified as potential clinical indicators of allograft injury and rejection. These biomarkers provide valuable insights into the immunopathology of nephron injury and have the potential to improve overall outcomes in post-RT patients. However, the clinical application of biomarkers faces challenges such as sensitivity, specificity, and inter-observational variability. Extensive validation studies and assay standardization are necessary before biomarkers can be confidently integrated into clinical practice. Furthermore, statistical limitations and the variability of transplant recipients’ clinical course must be addressed to generate robust evidence. For now, more scientific research is needed to fully harness the potential of biomarkers in guiding personalized care for RT patients.

## Figures and Tables

**Table 1:** Biomarkers for monitoring nephron damage post-RT. NB: ↑—increased; ↓—decreased. Adapted after Kępka et al. [10].

	Biomarker	Clinical Evidence
**Renal Corpuscle**	**Urea/** **Creatinine**	The oldest biomarkers of glomerular injury [42], **urea** (protein metabolism) and **creatinine** (muscular metabolism) are both byproducts filtered by the glomeruli and excreted in urine. ↑ serum urea/creatinine, i.e., nitrogen retention, are indicative of ↓ GFR and renal dysfunction.
	**Cystatin C (CYC)**	**CYC** = Low-molecular-weight cysteine protease inhibitor [43], secreted by all nucleated cells at a constant rate, freely filtered by the glomerulus and completely reabsorbed by proximal tubule cells, i.e., negligible amounts in final urine [44]. Normal range of 0.05–10.47 mg/L [45]. ↑ urinary levels may indicate proximal tubule dysfunction and tubule-interstitial disease [39].
	**Proteinuria**	**Proteinuria** is caused by ↑ filtration of plasma proteins and ↓ proximal tubular reabsorption [46,47]. Levels ≥ 0.5 g/24 h = independent risk factor for progressive tubular-interstitial fibrosis and a strong predictor of ESRD [48,49,50,51]. It may indicate established renal damage associated with ↓ GFR [52]. Post-RT, signals poor renal graft function and potential graft failure [53].
	**Albuminuria**	Urinary albumin (the main plasmatic protein) is a more sensitive marker of GFR than proteinuria [54]. **Micro-albuminuria** (<200 μg/min) is a better indicator of kidney transplant condition than proteinuria [55]. It predicts renal graft loss and offers early detection of allograft changes [56], but also reflects both glomerular and chronic allograft damage and may be indicative of interstitial inflammation [57]. Thus, it is also considered a predictor of long-term allograft outcomes in RT recipients. **Urine albumin-to-creatinine ratios** (UACRs) are also recommended for post-RT monitoring [57].
	**Adhesion Molecules** **(between Podocytes & Basal Membranes)**	**Integrins** = transmembranary glycoproteins with two subunits, α and β, which promote cellular attachment/migration/invasion, along the surrounding extracellular matrix (ECM), and are necessary to maintain cellular survival and functions. The β1 subtypes = major class of cell substrate receptors, specifically binding collagens, laminins, and fibronectins [58]. **Integrin α3/β3** are particularly recommended for monitoring the function of allografts, both in the early and long-term setting, after RT [58].
		Vascular cell adhesion molecule-1 (**VCAM-1**), Soluble vascular cell adhesion molecule 1 (**sVCAM-1**)/(CD106), and Anti-intercellular adhesion molecule-1 (**ICAM-1**), as members of the immunoglobulin (Ig) superfamily, are the chief endothelial cell proteins recognized by white cell integrins [10]. ↑ urinary sVCAM-1, interleukin (IL)6, sIL6R, and tumor necrosis factor (TNF)R1 levels in the first 2 weeks post RT indicate AR [59]. ↑ urinary sICAM-1 levels have also been reported in AR patients [60]. RT patients with proteinuria showed ↑ sVCAM/sICAM urinary levels [61]. Currently, the determination of cell adhesion molecules is recommended as a non-invasive monitoring tool for AR post RT [62].
**Proximal Tubules**	**α1-Microglobulin (α1M)**	**α1M =** 27 kDa glycoprotein from the lipocalin family, structurally related to retinol binding protein (RBP), synthesized by liver cells, with various functions, i.e., immunoregulation by binding to T and B lymphocytes, and involvement in heme-complex catabolism [39,62,63]. Stability in acidic urine makes it a sensitive indicator of proximal tubule renal damage, i.e., ↑ urinary levels may be a consequence of GF deterioration. An ↑ α1M/creatinine ratio = an early and sensitive indicator of poor allograft function/prognosis/long-term survival after RT [64], i.e., 6 months post RT, 32% had microalbuminuria.
	**β2-Microglobulin (β2M)**	**β2M =** 11.8 kDa protein, part of the major histocompatibility complex (MHC) class I molecules, found on the surface of all nucleated cells [63]. It undergoes GF and then is reabsorbed and catabolized in the proximal tubules. β2M excretion is typically used to evaluate nephrotoxic damage, such as that caused by aminoglycoside antibiotics or heavy metal salts. Urinary β2M can be helpful in evaluating the state of a transplanted kidney, yet the interpretation of results should be done with caution due to the variety of factors that can influence β2M plasmatic/urinary concentration, renal filtration ability, and tubular function, i.e., drugs, ischemia–reperfusion complications, or true renal graft rejection [64].
	**Retinol binding protein (RBP)**	**RBP =** 21 kDa protein belonging to the lipocalin family, primarily synthesized in the liver, it mainly transports retinol (vitamin A) from the liver to peripheral tissues [63,64,65,66]. RBP is filtered by the glomerulus, and then reabsorbed and catabolized in proximal tubules. ↑ urinary RBP can be a result of impaired GF and/or reabsorption in the renal proximal tubules. Due to its greater stability in acidic urine, RBP is considered a better biomarker for proximal tubule damage than β2M [10].
	**Brush Border Tubular Enzymes** **(↑urinary Excretion = Tubular Brush Border Membrane Damage/Microvillus Loss [29])**	**Adenosine deaminase binding protein (ABP)** = 120 kDa glycoprotein found in various tissues such as lungs, liver, placenta, and brush border of renal proximal tubules. It is involved in the regulation of adenosine levels and has been implicated in several physiological and pathological processes [67]. ↑ urinary ABP is considered an early indicator of AKI and has been reported in various clinical situations, such as ischemia without sepsis, RT, toxic renal tubular damage, and neonatal sepsis. Some researchers have suggested that ABP may be the best marker of acute renal damage, even better than β2M or α1M [67]. Due to higher ABP excretion in RT recipients compared to those with normal renal function, ABP is a good indicator for detecting graft failure [68].
		**Alkaline phosphatase (AP) =** 140 kDa membrane-bound glycoprotein found in various tissues, including renal proximal tubular structures. AP is involved in the metabolism of organic phosphates [69]. One common reason for declining function in allografts post-RT is the nephrotoxicity of chronic immunosuppressive therapy. ↑ urinary AP levels can be a sign of renal damage due to the use of immunosuppressive drugs, i.e., usually Cyclosporine A [69].
		**γ-glutamyl-transferase (GGT) =** ubiquitous enzyme found in the cell membranes of numerous tissues such as kidneys, bile duct, pancreas, gallbladder, spleen, heart, brain, and seminal vesicles. It plays an integral role in amino acid transport across the cell membrane and in the metabolism of leukotrienes. Notably, GGT is also involved in maintaining the balance of oxidative stress within the cell by participating in glutathione metabolism. ↑ urinary GGT provides reliable evidence of nephrotoxicity, such as that caused by prolonged use of anti-rejection drugs in RT patients. An absence of GGT/enzymes in urine suggests a return to normal function of the renal tubules [65].
		**Alanyl-aminopeptidase (AAP)**, an enzyme that degrades oligopeptides, when ↑ in urine, is associated with severe conditions such as acute renal tubular necrosis, rejection of renal graft, or the toxic effects of immunosuppressive drugs [39,65,70].
	**Cytosolic/Lysosomal Tubular Enzymes**	**α-/π-Glutathione-S-transferase (α-/π-GST)** = a specific cytosolic enzyme of tubular epithelial cells, which consists of two main isoenzymes: **α-GST** that thrives in alkaline pH, and **π-GST** which prefers an acidic pH. The α-GST is found in the epithelium of proximal tubular cells, and the π-GST in distal tubules [71]. The determination of α-GST and π-GST in urine is utilized for diagnosing acute renal graft rejection with acute tubular necrosis [65]. A differentiated increase in the urinary excretion of α-GST vs. π-GST may indicate the location of nephron damage [45,71,72,73,74,75].
		**N-acetyl-β-D-hexosaminidase (HEX)** = a lysosomal renal enzyme and one of the most commonly determined urinary markers for tubular damage, i.e., HEX activity increases early on, prior to the onset of disturbances in renal excretion. Mainly found in proximal tubular cells, HEX is thus specific, i.e., ↑ molecular weight (>130 kDa) prevents glomerular filtration [76,77]. During active kidney disease, HEX activity consistently rises. ↑ urinary activity of HEX/its isoenzyme HEXB indicates damage to renal tubular cellularity. Thus, urinary HEX and, particularly, HEXB, may serve as specific markers for proximal tubular damage post-RT [76,77].
		**Fructose-1,6-bisphosphatase (FBP-1,6)** = primarily localized in the convoluted and to a lesser extent in the straight portion of proximal renal tubules. Similar to HEX and GST, it indicates the precise location of allograft nephron damage [73,78]. ↑urinary FBP-1,6 was observed post-RT. Urinary FBP-1,6 excretion was significantly lower in patients with a median cold ischemia time of <22 h, compared to those with >22 h. Even in the absence of graft dysfunction, if the cold ischemia period is extended, urinary excretion of FBP-1,6 correlates with the extent of damage to the renal tubules [79]
**Distal Tubules**	**Urinary** **Osmolality**	**Urine osmolality** refers to the concentration of solutes in urine, and is regulated by the activity of antidiuretic hormone (ADH) in the distal nephron [80]. An important parameter for evaluating the function of distal renal tubules. ↓ urinary osmolality suggests the presence of distal tubular dysfunction [80].
	**Tamm-Horsfall Glycoprotein (THP)**	**THP**, i.e., uromodulin = protein synthesized by renal tubular cells in the thick ascending limb of Henle’s loop and the distal convoluted tubule. THP is the most abundant protein in normal urine, and its concentration is directly proportional to the number of functioning nephrons [81]. A ↓ THP excretion is a sensitive indicator of tubular dysfunction in patients with CKD [81].
	**Renal** **Kallikrein**	**Renal kallikrein** = an enzyme that regulates blood pressure and sodium excretion in the kidney [82]. Urinary kallikrein is considered a sensitive marker of distal tubular dysfunction, and its levels have been shown to decrease in various types of renal disease [82].
	**Annexin A11 (ANX11)**	**ANX11** = a calcium-binding protein that is found in high quantities in distal tubular cells and glomerular epithelium. ANX11 has been identified as a useful marker of acute and chronic renal graft rejection [58].
	**Renal Papillary Antigen** **(RPA)-1**	**RPA-1** = a sensitive and specific antigen of renal papillary cells, i.e., a useful marker of damage to renal collecting tubules. RPA-1 has been shown to be a sensitive and specific urinary marker of renal papillary cell injury in both animal models and humans [75].
	**Prominin-2 (PROM-2)**	**PROM-2** = a cellular membrane glycoprotein (112 kDa), with peak expression in epithelial cells of fully developed kidneys, i.e., a cholesterol-binding protein, associated with apical and basolateral plasmalemma protrusions in polarized renal epithelial cells that is released into urine [83]. PROM-2 has been identified as a novel biomarker, specific for distal tubules and collecting ducts, in human and murine kidneys, useful biomarker for the functional assessment of distal renal tubules [84].
	**μ-Glutathione-S-Transferase (μ-GST)**	**μ-GST** = a conjugating glutathione present in tubular epithelial cells, i.e., mainly the ascending part of Henle’s loop [75], alongside π-GST. It represents a nephrotoxicity-specific biomarker. μ-GST is an early biomarker for Henle’s loop and distal tubule damage, and has been shown to be more specific than albuminuria for assessing nephrotoxicity [85]. ↑ urinary μ-GST levels can be observed in response to treatment with nephrotoxic drugs, such as cisplatin.

**Table 2:** Summary of evidence regarding emerging predictive biomarkers for IRI/DGF [98,99,100,101,102,103,104,105,106,107,108,109,110,111,112,113,114,115,116,117].

		Pre-RT Applications	Post-RT Applications
**Ischemia–reperfusoin Injury (IRI)/Delayed Graft Function (DGF)**	**Proteomic Data**	**Donor urinary biomarkers**: → **NGAL, KIM-1**, and fatty acid binding protein (**FABP**) levels have predictive value for DGF [98,99,100]; **Recipient cytokines**: → Plasma levels of soluble interleukin 6 receptor (sIL-6R) and low soluble gp130 correlate with DGF [101]; **Recipient circulating regulatory T-cells**: → Expressing tumor necrosis factor receptor 2 (TNFR-2), as a peripheral blood DGF predictor [102].	**Recipient urinary biomarkers** **NGAL, KIM-1, IL-18 and FABP** are specific for AKI and/or IRI [103,104], and are related to renal allograft dysfunction [105,106,107,108,109]: NGAL/KIM-1 correlate with DGF severity [76]; NGAL is associated with long-term graft dysfunction, and is predictive of DGF [110]; IL-18 can predict DGF within 4 h post RT [111].
	**Genomic/Transcriptomic Data**	**Predictive of DGF on pre-RT allograft biopsy samples**: **Adhesion molecules**: ICAM-1 upregulation in tubular epithelial cells (only cadaveric grafts) [112]; **Anti-apoptotic genes**, i.e., B-cell lymphoma 2 (Bcl-2)/extra-large (Bcl-xl): lack of upregulation in donor renal tissue [113]; **Complement system, metabolic and immune pathway genes**: allograft upregulation [114]; **↑ lipocalin-2 (LCN)/NGAL [115]**; **↑ cyclin-dependent kinase inhibitor 2A (CDKN2A) [116]**.	**MicroRNAs (miRNAs)** = short endogenous non-coding RNAs that inhibit gene expression; **miR-182-5p and mi-21-3p**, have been found to play a role in the pathogenesis of DGF [117]. **Secretory Leukocyte Peptidase Inhibitor (SLPI)**: ↑ urine and serum transcript expression levels were reported in post-RT AKI cases [117].

NB: ↑—increased.

**Table 3:** Summary of evidence regarding emerging post-RT biomarkers for AR [4,131,132,139,140,141,142,143,144,145,146,147,148,149,150,151,152,153,154,155,156,157,158,159,160,161,162,163].

	Postoperative Biomarkers Specific for Acute Renal Allograft Rejection
Proteomic Evidence	**Plasmatic samples:** ➢ Twelve diagnostic proteins, with a fold-change in ≥1.15, in RT patients with biopsy-confirmed AR, were identified: - Seven proteins with ↑ levels: titin (TTN), lipopolysaccharide-binding protein (LBP), peptidase inhibitor 16 (PI16), complement factor D (CFD), mannose-binding lectin 2 (MBL2), protein Z-dependent protease, and β2M; - Five proteins with ↓ levels: kininogen-1, afamin, serine protease inhibitor, phosphatidylcholine-sterol acyltransferase, and sex hormone-binding globulin [139]; ➢ ↑ Nuclear Factor Kappa B (NFκB), Signal Transducer and Activator of Transcription 1 (STAT1), STAT3, and 63 other proteins showed ≥2-fold differences in expression levels between 13 RT patients, with vs. without AR [140]; ➢ ↑ α-1 antitrypsin (A1AT), α-2 antiplasmin (A2AP) and serum amyloid A (SAA) expression in confirmed AR cases (31 RT cases in total), and apo-lipoprotein CIII (APOC3) expression was exclusive to AR cases [141].
	**Urinary samples:** ➢ Uromodulin (THP), Pigment Epithelium-Derived Factor (PEDF/SERPINF1), and CD44 were indicative of AR in a 60 RT case cohort [142]; ➢ Insulin-Like Growth Factor Binding Protein 7 (IGFBP7), Vasorin (VASN), Epidermal Growth Factor (EGF), and Galectin-3-Binding Protein (LG3BP) levels were found to be ↑ in 12 RT patients with AR [143]; ➢ Actin β (ACTβ), Dipeptidyl-Peptidase 4 (DPP4), Fibrinogen α Chain Precursor (FGα), Fibrinogen β Chain Precursor (FGβ), Fibrinogen γ (FGγ), Histone Cluster 1 H4 Family Member B (HIST1H4B), HLA class II protein HLA-DRB1 (HLA-DRB1), Keratin 7 (KRT7), KRT14 levels were highly specific for AR, with significant differences within this subgroup, i.e., a fold-increase >1.5, as compared to all others (154 RT cases in total) [144].
Transcriptomic Evidence	**Messenger (m)RNAs:** ➢ ↑ Granzyme B (GZMB), perforin (PRF1) and Fas Ligand (FASLG) mRNA levels, in peripheral blood samples and graft tissue biopsies, among 25 AR cases [145]; ➢ ↑ urinary GZMB/PRF1 mRNA levels in 22 post-RT patients with AR [146]; ➢ ↑ OX40, OX40L, programmed cell-death (PD)-1 and Forkhead box P3 (FOXP3) mRNA levels in urinary cellularity strongly predict acute kidney transplant rejection, i.e., upregulated AR-specific gene signature in 21 RT cases. Additionally, OX40, OX40L, and Foxp3 mRNA levels predicted reversal of AR, while OX40 alone predicted graft loss post-AR [147]; ➢ ↑ Foxp3 mRNA urinary levels were found to be AR-specific, when comparing 36 confirmed AR cases with CAN/normal biopsy cohorts [148]; ➢ The Clinical Trials in Organ Transplantation (CTOT)-04 multicenter trial (485 post-RT patients): a three-gene AR-specific urinary signature was reported for biopsy-confirmed AR cases, i.e., ↑ CD3ε mRNA, IFN-inducible protein 10 (IP-10/CXCL10) mRNA, and 18S ribosomal RNA [149]; ➢ CTOT-01 multicenter study (280 post-RT patients): ↑ urinary CXCL9 mRNA represents the best predictor of AR [131], whereas ↓ levels may be indicative of a proportionally ↓ risk of immunological allograft complications [132].
	**MicroRNAs (miRNAs):** ➢ ↑ miR-181a, miR-483-5p, and miR-557 expression levels, in the serum of 15 post-RT patients, were found to be significant predictive factors for AR [150]; ➢ Urinary miR-210 expression is ↓ during AR, but normalizes after successful treatment (62 AR cases) [151]; ➢ Serum levels of miR-223 and miRNA10a were shown to be significantly ↓ during AR (12 RT cases) [152]; ➢ Inhibition of miR-155 and miR-221 is associated with T-cell proliferation, whereas miR-142-3p is associated with tolerant kidney allograft recipients [153].
Genomic Evidence	**Gene signatures** (array technology on multicenter graft biopsies and paired peripheral blood samples): ➢ **SNSO1 clinical trial dataset** (367 pediatric RT patients) [154]—A five-gene panel proved to accurately identify patients affected by AR, using microarray with quantitative polymerase chain reaction (qPCR) for confirmation, namely: **DUSP1**, **NKTR**, **MAPK9**, **PBEF1** and **PSEN1** [155,156]; ➢ **AART study** (436 adult/pediatric RT patients) [157]—An integrative AR screening panel of 43 rejection-related genes was compiled and assessed in RT recipients, through whole-genome microarray analysis. These genes were selected based on the various principles [155,158], and showed the following results: ○ An initial 10-gene panel (**CFLAR, DUSP1, IFNGR1, ITGAX, MAPK9, NAMPT, NKTR, PSEN1, RNF130, RYBP**), which had previously been found to be significantly associated with AR in pediatric RT patients, i.e., cross-validated by the SNSO1 randomized multicenter trial [154], correctly predicted AR in 87.4% of adult samples, with 93.8% specificity and 74.5% sensitivity; ○ Out of the 43 genes tested, 31 were differentially expressed in adult RT recipients with AR. Eight of these genes were part of the initial 10-gene panel [159]. In the adult dataset, a subset of 15 out of the 43 genes accurately classified 91.6% of the AR and non-AR samples [157]. However, this approach failed to identify AR in a purely pediatric SNSO1 data subset [154,159]. To ensure the test could identify AR regardless of patient age, the original panel of 10 genes was retained, and 7 additional genes (**CEACAM4, EPOR, GZMK, RARA, RHEB, RXRA, SLC25A37**) were added to optimize the gene set’s performance for discriminating AR in both adult and pediatric samples [157]. ○ The resulting optimized 17-gene panel, constituting the Kidney Solid Organ Response Test (**kSORT**), was used successfully to detect RT patients at high risk for AR, demonstrating better diagnostic accuracy compared to the initial 10-gene panel [157] and apparently being able to predict rejection episodes up to 3 months prior to their clinical detection by the current gold standard methods (biopsy) [160]. ➢ **CRM meta-analysis [161]**: A common rejection module (CRM), consisting of 11 AR-specific genes, was identified through a meta-analysis of eight independent transplant patient datasets, i.e., 236 graft biopsy tissue samples, from four different organs (heart, kidney, liver, and lung), namely: **BASP1, CD6, CXCL10, CXCL9, INPP5D, ISG20, LCK, NKG7, PSMB9, RUNX3, TAP1**. These genes were found to be significantly overexpressed in AR-confirmed cases, across all the transplanted organs within the study population. Importantly, this initial high diagnostic capability for AR reported for the CRM, was further validated within this meta-analysis, on 5 different additional transplantation cohorts (945 samples), demonstrating both high specificity and sensitivity when tested. Moreover, in these additional cohorts, CRM genes showed a correlation with the extent of graft injury and also displayed predictive capabilities regarding future graft injury occurrence, as determined by protocol biopsies [161]. Furthermore, external validation initiatives have corroborated the aforementioned results on post-RT patients, as follows: ○ The tissue 11-gene CRM (**tCRM**) qPCR score was found to be significantly ↑ in AR, with the greatest significance for CXCL9/10. It was also correlated with the extent of AR lesions and was predictive of CAD [162]. ○ A recent study evaluated the CRM gene set in the urine of RT patients with acute allograft dysfunction, and found that only 5 out of the 11 genes were highly significant at the time of rejection [163]. However, the urinary common rejection module (**uCRM**) score was also found to be elevated in other kidney injuries, such as acute tubular necrosis and BK virus nephropathy (BKVN) [163].

NB: ↑—increased; ↓—decreased; AART = Assessment of Acute Rejection in Renal Transplantation; BASP1 = Brain abundant membrane attached signal protein 1-5p15.1; CD6 = CD6 molecule-11q12.2; CEACAM4 = Carcinoembryonic antigen-related cell adhesion molecule 4-19q13.2; CFLAR = CASP8 and FADD-like apoptosis regulator gene-2q33.1; DUSP1 = Dual-specificity phosphatase 1–5q35.1; EPOR = Erythropoietin receptor encoding gene-19p13.2; GZMK = Granzyme K encoding gene-5q11.2; IFNGR1 = Ligand binding chain of the gamma interferon receptor gene-6q23.3; INPP5D = Inositol polyphosphate-5-phosphatase D-2q37.1; ISG20 = IFN-stimulated exonuclease gene 20-15q26.1; ITGAX = Integrin α-X-chain protein-16p11.2; LCK = LCK proto-oncogene, SRC family tyrosine kinase-1p35.2; MAPK9 = Mitogen-activated protein kinase 9-5q35.3; NAMPT = Nicotinamide phosphoribosyl-transferase-7q22.3; NKG7 = Natural killer cell granule protein 7-19q13.41; NKTR = Natural killer cell triggering receptor-3p22.1; PSMB9 = Proteasome subunit beta 9-6p21.32; PSEN1 = Presenilin 1-14q24.2; RARA = Retinoic acid receptor-17q21.2; RHEB = Ras homolog enriched in brain-7q36.1; RNF130 = Ring finger motif-5q35.3; RUNX3 = Runt related transcription factor 3-1p36.11; RYBP = RING1 and YY1 binding protein-3p13; RXRA = Retinoic X receptor α-9q34.2; SLC25A37 = Solute carrier family 25 number 37-8p21.2; TAP1 = Transporter 1, ATP binding cassette subfamily B member-6p21.32.

## Data Availability

The data presented in this study are available on request from the main author.
